# Deciphering the Bacterial Microbiome of Citrus Plants in Response to ‘*Candidatus* Liberibacter asiaticus’-Infection and Antibiotic Treatments

**DOI:** 10.1371/journal.pone.0076331

**Published:** 2013-11-08

**Authors:** Muqing Zhang, Charles A. Powell, Lesley S. Benyon, Hui Zhou, Yongping Duan

**Affiliations:** 1 Indian River Research and Education Center, IFAS-UF, Fort Pierce, Florida, United States of America; 2 USDA-ARS, US Horticultural Lab, Fort Pierce, Florida, United States of America; 3 State Key Lab for Conservation and Utilization of Subtropical Agro-bioresources, Guangxi University, Nanning, Guangxi, China; International Atomic Energy Agency, Austria

## Abstract

The bacterial microbiomes of citrus plants were characterized in response to ‘*Candidatus* Liberibacter asiaticus’ (Las)-infection and treatments with ampicillin (Amp) and gentamicin (Gm) by Phylochip-based metagenomics. The results revealed that 7,407 of over 50,000 known Operational Taxonomic Units (OTUs) in 53 phyla were detected in citrus leaf midribs using the PhyloChip™ G3 array, of which five phyla were dominant, *Proteobacteria* (38.7%), *Firmicutes* (29.0%), *Actinobacteria* (16.1%), *Bacteroidetes* (6.2%) and *Cyanobacteria* (2.3%). The OTU62806, representing ‘*Candidatus* Liberibacter’, was present with a high titer in the plants graft-inoculated with Las-infected scions treated with Gm at 100 mg/L and in the water-treated control (CK_1_). However, the Las bacterium was not detected in the plants graft-inoculated with Las-infected scions treated with Amp at 1.0 g/L or in plants graft-inoculated with Las-free scions (CK_2_). The PhyloChip array demonstrated that more OTUs, at a higher abundance, were detected in the Gm-treated plants than in the other treatment and the controls. Pairwise comparisons indicated that 23 OTUs from the *Achromobacter* spp. and 12 OTUs from the *Methylobacterium* spp. were more abundant in CK_2_ and CK_1_, respectively. Ten abundant OTUs from the *Stenotrophomonas* spp. were detected only in the Amp-treatment. These results provide new insights into microbial communities that may be associated with the progression of citrus huanglongbing (HLB) and the potential effects of antibiotics on the disease and microbial ecology.

## Introduction

Huanglongbing (HLB), the most devastating citrus disease worldwide, is vectored by phloem-feeding insects and caused by fastidious bacterial pathogens (*Candidatus* Liberibacter) [1], [2], [3]. The three species of the pathogen have been identified by their 16S rRNA sequences: *Candidatus* Liberibacter asiaticus (Las), the most prevalent species in Asia and America [4], [5], *Candidatus* Liberibacter africanus (Laf) in Africa [6], and *Candidatus* Liberibacter americanus (Lam) in South America [7]. In the U.S., citrus HLB was first discovered in August of 2005 in South Florida. Currently, HLB exists in all 34 citrus-producing counties in Florida and has caused an estimated $3.63 billion in lost revenues and 6,611 lost jobs by reducing orange juice production [8]. To date, there is no effective strategy to control citrus HLB after it is established [5], [9].

Soon after a bacterium was reported to be associated with HLB, antibiotics were first used to control the pathogen. Different types of antibiotics, such as tetracycline and penicillin, were injected into infected citrus trees to temporarily relieve HLB symptoms and decrease Las bacterial titers [10]. Injecting antibiotics was recommended as a part of the integrated management program in India [11]. In our previous studies, different kinds of antibiotics were tested for efficacy against the HLB bacterium while assessing their phytotoxicity to citrus. A combination of a Beta-lactam antibiotic, penicillin, and an aminoglycoside, streptomycin, has been shown to act synergistically against the bacterium and facilitate the aminoglycoside's uptake, which leads to bacterial cell death [12], [13]. The microbial community structure in Las-infected field citrus plants treated with the above antibiotic combination has been analyzed. Our previous data detected 7,028 known Operational Taxonomic Units (OTUs) in citrus leaf midribs using the PhyloChip™ G3 array, of which *Proteobacteria* was constantly the dominant bacterial phylum, with the Alphaproteobacterial and the Gammaproteobacterial classes vying for prevalence. Bacterial cells in close proximity may be able to modify their microenvironment, making the composition of the microbial community an important factor in the ability of Las to cause HLB progression [14]. The microbial diversity associated with citrus HLB *in planta* has also been reported by other research groups [15], [16], [17]. Some plant growth-promoting bacteria, such as *Bacillus* and *Burkholderia*, were detected in non-infected leaf samples [15], while bacteria such as *Methylobacterium* and *Sphingobacterium* were present in root samples from HLB-affected trees [16].

A powerful oligonucleotide microarray of high-density 16S rRNA genes, the PhyloChip microarray, has been developed and effectively used to study bacterial diversity, especially from environmental samples [18]. In this article, we aim to decipher the bacterial microbiome in HLB-affected citrus versus non-infected citrus as well as in citrus plants treated with ampicillin and gentamicin using PhyloChip-based metagenomics.

## Materials and Methods

### Plant materials and treatments

HLB-affected budsticks were sampled from severely HLB-affected field rough lemons (*Citrus limonum* ‘Lemon #76’) at the USDA-ARS-USHRL farm in Fort Pierce, FL and tested positive for Las by real-time qPCR. They were soaked in the antibiotic treatments; ampicillin sodium at a concentration of 1.0 g/L (Amp, Sigma-Aldrich, St. Louis, MO) or gentamicin sulfate at a concentration of 100 mg/L (Gm, Sigma-Aldrich, St. Louis, MO) and water as the diseased control (CK_1_), overnight in a fume hood under ventilation and lighting. Las-free budsticks, which tested negative by qPCR from healthy rough lemons, were also soaked in water as the healthy control (CK_2_). The budsticks were grafted onto two-year-old healthy grapefruit (*Citrus paradisi* ‘Duncan’) rootstocks and covered using plastic tape for three weeks. Each experiment was replicated for three times with 45 scions. To improve scion growth, new flush from the rootstocks was removed after grafting and then allowed to grow. All experimental plants were grown in an insect-proof greenhouse. The first leaf samples from scions (rough lemon) and rootstocks (grapefruit) for DNA extraction were taken four months after inoculation, and second samplings were taken at six month after inoculation. The leaves were washed in tap water and then rinsed three times with sterile water. The midribs of the leaves were excised, frozen in liquid nitrogen, and stored at −80°C. The midribs of five leaves from each sample were pooled, and DNA was isolated for qPCR analysis as described previously [13], [19]. The scion growth rate (%) was defined as the number of scions that had newly emerging leaves or flushes out of the total grafted scions. The scion infection rate (%) was defined as the number of Las infected scions with threshold cycle (Ct) values lower than 32.0 out of the total grafted scion number. The Las transmission rate (%) was defined as the number of the grafted plants' rootstocks that tested Las positive by qPCR with Ct values less than 32.0. Data were analyzed by a generalized linear mixed model using the SAS procedure GLIMMIX. Differences among treatment levels were determined with the LINES option of the LSMEANS statement.

### PCR amplification of 16S rRNA genes

DNA for the PhyloChip™ G3 analysis, which was extracted from all scion samples of the same treatment, was pooled in equal amounts and quantified by the PicoGreen® method. PCR amplifications of 16S rRNA genes were conducted as described previously [14].

### PhyloChip™ G3 analysis

The PhyloChip™ G3 analysis was conducted by Second Genome Inc. (San Bruno, CA). The 16S rRNA amplicons and a mixture of amplicons at known concentrations (spike-mix) were combined, fragmented using DNAse1 (Invitrogen, Carlsbad, CA), and biotin-labeled using the recommended protocol for Affymetrix Prokaryotic Arrays. Labeled products were hybridized with three replicates overnight at 48°C at 60 rpm. The arrays were washed, stained, and scanned as described in Hazen et al. (2010) [18].

### Data analyses

Preliminary data analyses were performed by Second Genome (San Bruno, CA) as described in Hazen et al. (2010) [18]. Briefly, to calculate the summary intensity for each feature on each array, the central nine pixels of individual features were ranked by intensity and the 75^th^ percentile was used. Probe intensities were background-subtracted and scaled to the PhyloChip™ Control Mix. Array fluorescence intensity was collected as integer values ranging from 0 to 65,536 (2^16^). Fluorescent intensities for sets of probes complementing an OTU were averaged after discarding the highest and lowest, and the means were log2 transformed. Thus, they are decimal numbers ranging from 0 to 16. For compatibility with some statistical operations, the HybScore was multiplied by 1,000 and then rounded to the nearest integer allowing a range of 0 to 16,000. Thus, if an OTU's HybScore changes by 1,000, it indicates a doubling in the fluorescence intensity. An OTU is defined by a group of highly similar 16S rRNA gene sequences. In most OTUs, the intra-OTU similarity is >99%. The data was reduced to only the bacterial OTUs meeting criteria for confirmed presence as described in Hazen et al. (2010) [18]. After the OTUs were identified for inclusion in the analysis, the values used for each OTU-sample intersection were populated in two distinct ways: i) Abundance metrics were used directly (AT); ii) Binary metrics were created where 1's represent presence and 0's indicate absence (BT).

The HybScore was averaged from all present OTUs in a taxonomic family, such as *Alcaligenaceae*. The families containing more than 1% of the OTUs present were used for pairwise comparisons and construction of circular trees. The ratios were calculated as follows: 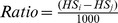, where HS represents the average HybScore of OTUs in each family, i represents one treatment and j represents another treatment. The five comparative trees, CK_1_ versus CK_2_, Amp versus CK_1_, Amp versus CK_2_, Gm versus CK_1_ and Gm versus CK_2_, were constructed by the NJ method in BioNJ [20], [21], and these were used as the initial trees for the maximum likelihood method in PhyML [22], [23]. The resulting phylogenetic trees were uploaded to the iTOL website (http://itol.embl.de/) and reconstructed as circular trees [24], [25]. The number of OTUs and their family ratios are presented in the circular trees.

## Results

### Bacterial microbiome in response to ampicillin and gentamicin

**Figure 1:**
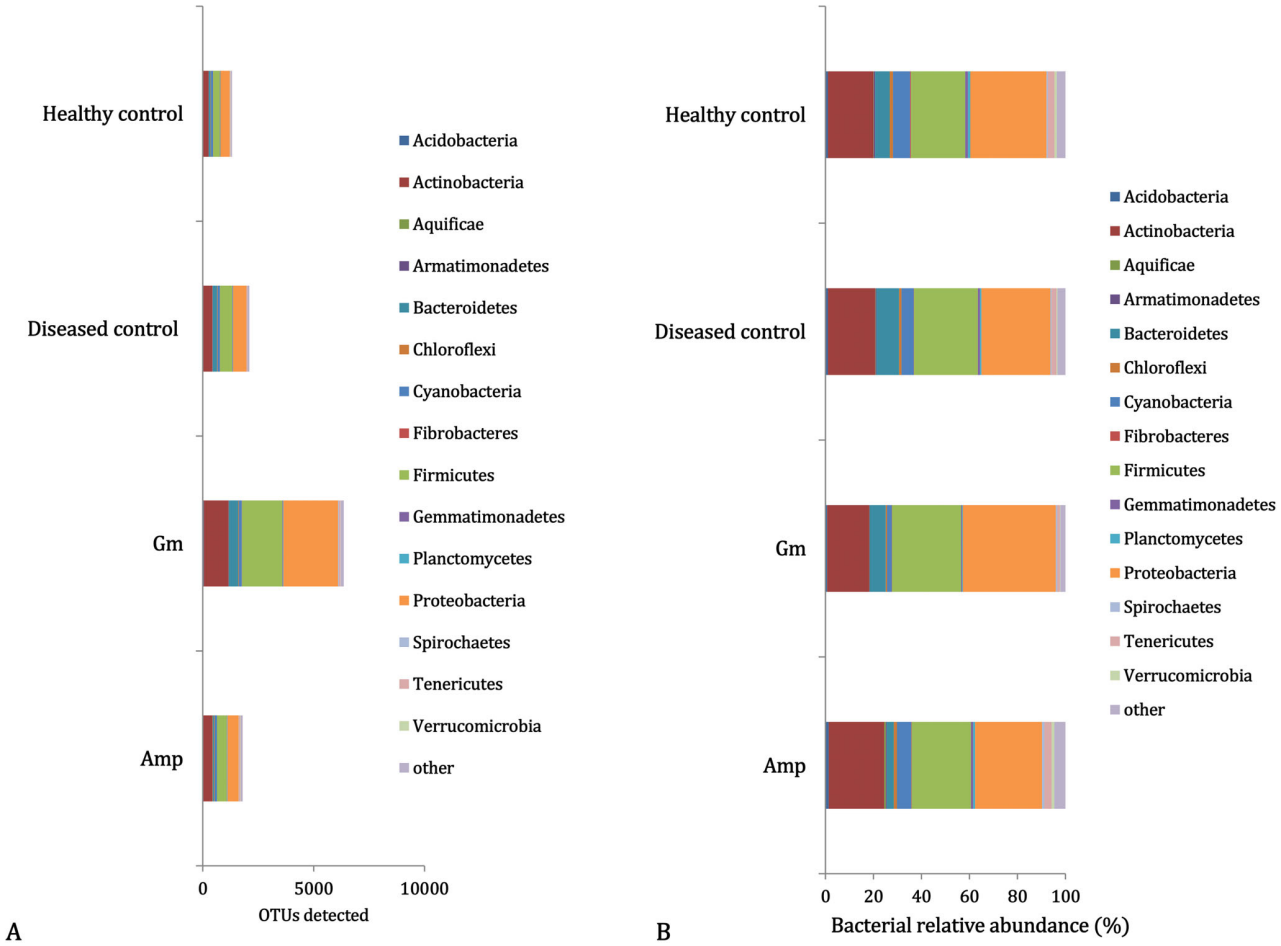
Bacterial community of leaf midribs of scions. **A**) Composition and **B**) relative abundance of the bacterial Operational Taxonomic Units (OTUs) present in leaf midribs of scions from grapefruit rootstocks grafted with HLB-affected lemon scions treated with ampicillin (Amp), gentamicin (Gm) and water (disease control; CK_1_). The healthy plants were grafted using Las-free lemon scions (healthy control; CK_2_).

Of more than 50,000 bacterial OTUs in the PhyloChip™ G3 array, 7,407 were detected in midribs from the tested citrus plants, of which 585 OTUs (7.90%) were shared by all samples. A total of 6,356 OTUs (85.8%) found in the Gm-treated samples were significantly more than the number of OTUs found in the ampicillin treatment (Amp, 1,795 OTUs, 24.2%), the disease control (CK_1_, 2,099 OTUs, 28.3%) and the healthy control (CK_2_, 1,306 OTUs, 17.6%). After subtracting the OTUs also shared in the controls (CK_1_ and CK_2_), 589 OTUs (32.8%) and 4,472 OTUs (70.4%) were detected in the Amp- and Gm-treatments, respectively (Fig. 1A and Table S1).

In total 53 phyla were detected, of which five phyla were comprised of more than 150 OTUs (more than 2% of the total detected OTUs), *Proteobacteria* (38.7%), *Firmicutes* (29.0%), *Actinobacteria* (16.1%), *Bacteroidetes* (6.2%) and *Cyanobacteria* (2.3%). The relative proportions of the above five dominant phyla differed between the antibiotic treatments. Higher percentages of *Proteobacteria*, *Firmicutes* and *Bacteroidetes* were detected in the Gm-treated samples than in the Amp-treated samples. However, higher percentages of *Actinobacteria* and *Cyanobacteria* occurred not in the Gm-treated samples but in the Amp-treated samples (Fig. 1B and Table S1).

**Figure 2:**
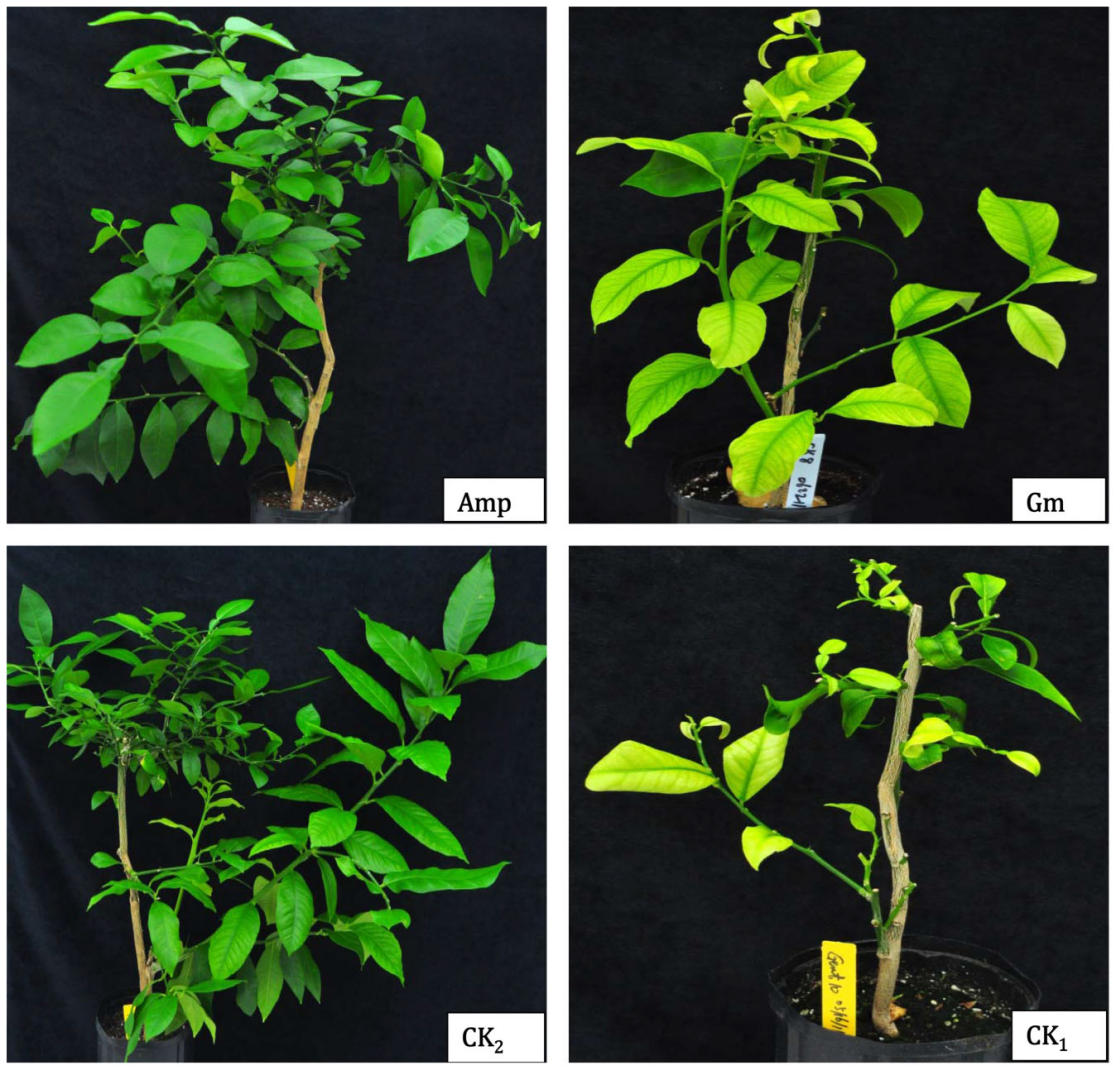
Huanglongbing symptoms of the inoculated plants. Healthy grapefruit rootstocks graft-inoculated with HLB-affected lemon scions treated with ampicillin at a concentration of 1.0 g/L (**Amp**), gentamicin at a concentration of 100 mg/L (**Gm**), and a water control (disease control, **CK_1_**); The healthy plants were graft-inoculated with Las-free lemon scions (healthy control, **CK_2_**).

Among the proteobacterial OTUs, the greatest numbers of unique OTUs were affiliated with Betaproteobacteria (17.0%), followed by Gammaproteobacteria (14.2%), Alphaproteobacteria (5.5%), Deltaproteobacteria (1.7%) and Epsilonproteobacteria (0.4%). Within the orders of Alphaproteobacteria, *Rhizobials*, to which the Las bacterium belongs, had the largest proportion, accounting for 2.1%, of the total detected OTUs, and this was due to their especially large percentages in the Gm-treatment and CK_1_. Within the Betaproteobacteria and Gammaproteobacteria, the families *Comamonadaceae* and *Pseudomonadaceae*, respectively, showed the highest OTU numbers and proportions in the Gm-treatment. The OTU62086, representing ‘*Ca.* Liberibacter’, was detected only in the inoculated plants from the Gm-treatment and the disease control (CK_1_), which showed typical HLB symptoms (Fig. 2).

### Antibiotic efficacy against Las bacterium and phytotoxicity to citrus

Amp and Gm were tested for their efficacy against the Las bacterium and evaluated for their phytotoxicity to citrus using scion growth rates. The Las-infected scions treated with Amp had >70% new growth as measured by emerging leaves or new flushes. However, only 47.5% and 50% of the scions had new growth when treated with Gm (Table 1) and water (disease control CK_1_), respectively. Variance analysis showed that there were significant effects of the antibiotic treatments (*Pr* = 0.000) on HLB bacterial titers, scion infection rates, and Las bacterial transmission rates in the fixed model. All graft-inoculated plants, whose HLB-affected scions were treated with Amp or whose scions were Las-free (CK_2_), tested negative for the Las bacterium via qPCR (Ct≈40.0), which indicates an estimated bacterial titer of <100 cells/g of plant tissue. No scions were infected and no Las bacteria were transmitted into the rootstocks, indicating that Amp successfully eliminated Las from the HLB-affected scions. The inoculated plants from the scions treated with Amp displayed normal growth, green leaves, and no HLB-like symptoms, similar to the plants grafted with Las-free scions (CK_2_) (Fig. 2). However, plants (scions and rootstocks) graft-inoculated using HLB-affected scions treated with Gm and water (CK_1_) had higher Las scion infection rates, transmission rates, and bacterial titers (approximate 1.4×10^6^ cells/g of plant tissue) (Table 1). The results indicate that Gm applied alone was not effective in eliminating the Las bacterium, and the plants showed typical HLB symptoms, such as yellow shoots and vein corking on leaves, in the rootstock (Fig. 2). The HybScore of OTU62086 in the Gm-treated samples indicated a fluorescent intensity greater than twice that measured in the Amp-treated samples and the healthy control (CK_2_) (Table 1).

**Table 1:** *Ca.* L. asiaticus (Las) and its transmission in grapefruit graft-inoculated with Las-infected lemon scions treated with ampicillin at 1.0 g/L (Amp), gentamicin at 100 mg/L (Gm), or water (disease control, CK_1_) as well as grafted with the Las-free lemon scions (healthy control, CK_2_).

Antibiotics	Scion survival (%)	Scion growth (%)	Scion infected (%)	Las transmission (%)	HybScore	Ct value	
						Scion	Rootstock
Amp	96.4	73.2	0±0 c§	0±0 b	9614	39.7±0.09 a	39.6±0.21 a
Gm	80	47.5	100±0 a	70±0 a	12756	25.1±2.23 b	29.5±1.24 b
Diseased (CK_1_)	91.7	50.0	55±7.1 b	95±7.1 a	10413	25.0±0.24 b	25.2±0.85 b
Healthy (CK_2_)	93.6	63.1	0±0 c	0±0 b	9490	39.7±0.13 a	39.8±0.28 a

§ Data were analyzed by a generalized linear mixed model using the SAS procedure GLIMMIX. Differences among treatment levels were determined with the LINES option of the LSMEANS statement. Different letter showed the significant difference at 0.05 levels (*Pr*≤0.05).

### Specific OTUs associated with the diseased status and the antibiotic treatments

**Figure 3:**
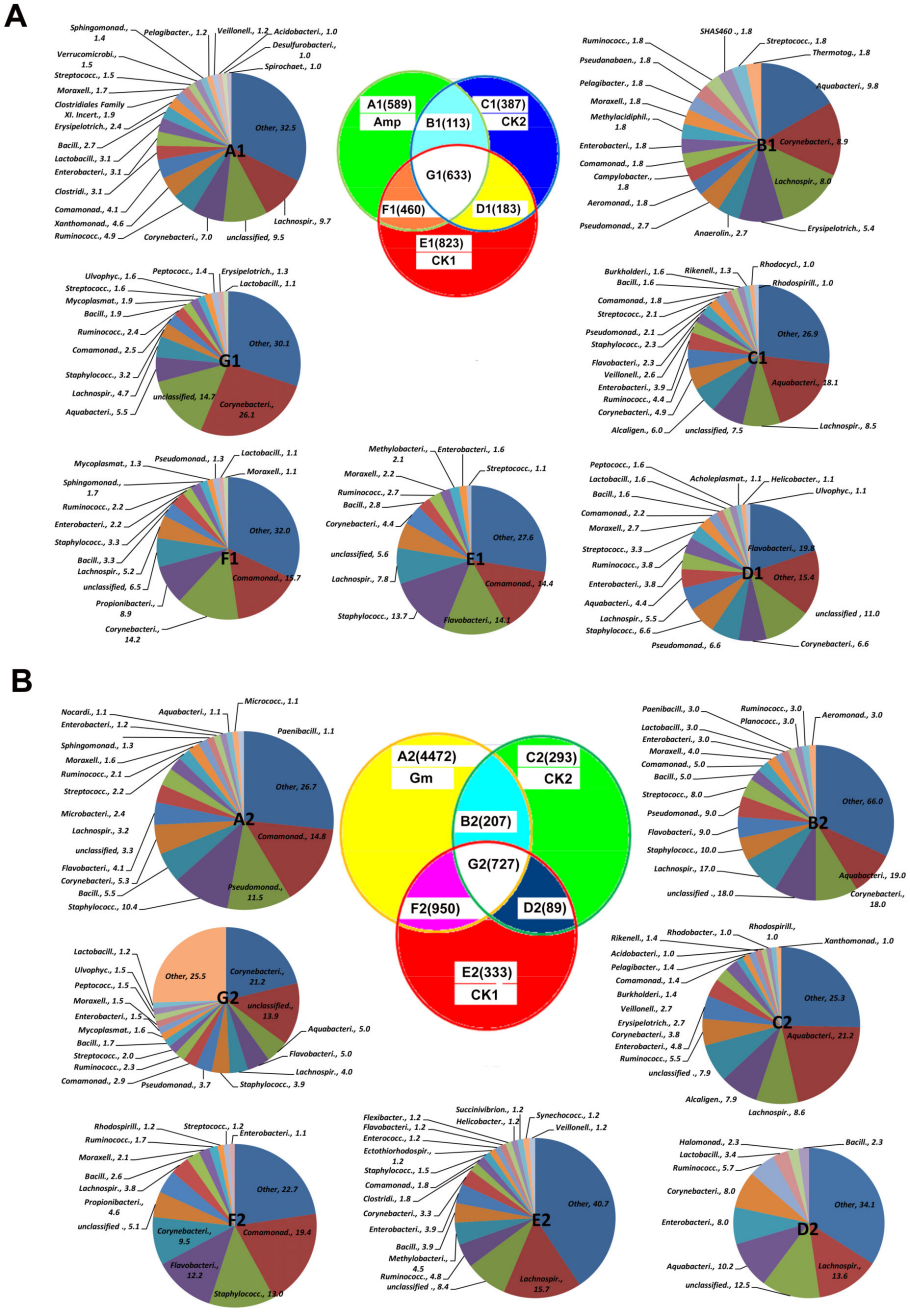
Distribution of the bacterial OTUs in response to antibiotic treatments. In the Venn diagram, the numbers in parentheses represent the number of bacterial OTUs that occurred in each antibiotic treatment [ampicillin (Amp) and gentamicin (Gm)], disease controls (CK_1_), healthy control (CK_2_) and their intersections. Pie charts A to G correspond to the appropriately labeled Venn diagram areas (A_1_ to G_1_ for the Amp treatment and A_2_ to G_2_ for the Gm treatment) and show families that contained over 1% of the total OTUs in each area. In pie charts A to G, the names of the families are followed by their frequencies as a percentage (%).

In a pairwise comparison of the disease (CK_1_) and healthy control (CK_2_), only 500 OTUs (total OTUs number in Fig. 3A-B_1_ and 3A-C_1_ or Fig. 3B-B_2_ and 3B-C_2_) in 114 families were present in the Las-free CK_2_ but absent in the Las-infected CK_1_, including 23 OTUs from *Alcaligenaceae* (only present in Fig. 3A-C_1_ or Fig. 3B-C_2_). However, 1,283 OTUs (total OTUs number in Fig. 3A-E_1_ and 3A-F_1_ or Fig. 3B-E_2_ and 3B-F_2_) in 155 families were present in CK_1_ but absent in CK_2_, including 190 OTUs from *Comamonadaceae*, 128 from *Staphylococcaceae* and 120 from *Flavobacteriaceae* (Fig. 3). When the abundance or hybridization scores (HybScores) of the detected OTUs was taken into account, we found that the relative abundance (ratio) of several bacterial OTUs is a more important indicator of disease status than the exclusive presence of specific bacterial OTUs. Circular trees comparing CK_2_ and CK_1_ showed that 18 families had more than 1% of the 500 OTUs detected in CK_2_ (Figure S1-B). However, only *Alcaligenaceae*, especially *Achromobacterxy losoxidans*, was more abundant in CK_2_ than in CK_1_. In CK_1_, 14 families had more than 1% of the total 1,283 OTUs detected and the families of *Methylobacteriaceae* and *Propionibacteriaceae* were more abundant (Fig. S1-A).

Plants graft-inoculated with HLB-affected scions treated with Amp appear Las-free and healthy. Our analyses in Fig. 3A showed that 18 out of 107 families contained over 1% of the 1,049 OTUs (total OTUs number in Fig. 3A-A_1_ and 3A-F_1_) detected in the Amp-treatment when compared to the healthy control (CK_2_), while 20 out of 132 families had over 1% of the 702 OTUs number in Fig. 3A-A_1_ and 3A-B_1_ detected when compared to the disease control (CK_1_). From the circular tree analysis, three families, *Xanthomonadaceae*, *Propionibacteriaceae* and *Cyanpbacteriaceae*, were abundant in the Amp-treatment (Fig. S3-E), while only *Alcaligenaceae* was abundant in CK_2_ (Fig. S3-F). However, two families, *Methylobacteriaceae* and *Staphylococcaceae*, had HybScores with doubled fluorescence intensities in CK_1_ (Fig. S2-D), while only *Xanthomonadaceae* was abundant in the Amp-treatment (Fig. S2-C).

Plants graft-inoculated with HLB-affected scions treated with Gm appeared diseased and contained a higher titer of the Las bacterium. Our analyses in Fig. 3B showed 18 out of 218 families had over 1% of the 4,679 OTUs (total OTUs number in Fig. 3B-A_2_ and 3B-B_2_) in the Gm-treatment when compared to CK_1_, while 15 out of 248 families had over 1% of the 5,422 OTUs (total OTUs number in Fig. 3B-A_2_ and 3B-F_2_) detected when compared to CK_2_. When compared to the disease control, all of the families, except *Lachnospiraceae* and *Ruminococcaceae*, were abundant in the Gm-treatment (Fig. S4-G), while only *Methylobacteriaceae* was more abundant in CK_1_ (Fig. S4-H). All families except *Ruminococcaceae* were abundant in the Gm-treatment (Fig. S5-I), while only *Alcaligenaceae* was more abundant at ratio of 4.0 in CK_2_ (Fig. S5-J).

## Discussion

A microbial community analysis provides an accelerated approach to understanding the structure and function of bacterial communities, and it may result inthe isolation and identification of novel bacteria [26]. This study provides a comprehensive survey of the richness and composition of microbial communities in the leaf midribs of HLB-affected citrus and healthy citrus plants as well as HLB-affected scions treated with antibiotics in greenhouse.

In a microbial community, more than 99% of the microorganisms have not been cultured [27], including the viable but nonculturable [28], [29] and the dormant [30]. The updated PhyloChip™ G3 effectively studies bacterial diversity and composition, and it is an improvement over the last version in a number of ways. These include an increase from 500,000 to 1.1 million probes, the inclusion of strain-specific probe sets, the ability to detect over 50,000 OTUs instead of ∼9,000 OTUs [18], and the utilization of over 320,000 sequences in the reference database, which is over 10 times greater than that for the PhyloChip™ G2. Many endophytic bacteria have been isolated from citrus [14], [15], [31]. Compared to the 15 phyla detected in citrus leaves in a previous report using PhyloChip™ G2 [15], we have detected 53 phyla in the HLB-affected citrus in greenhouse using the updated PhyloChip™ G3. A total of 7,407 bacterial OTUs were detected from the bacterial microbiome, of which 585 OTUs were present in all samples. A total of 6,356 OTUs were detected in the Gm-treatment, which was significantly higher than those detected in the Amp-treatment (Amp, 1,795 OTUs), the disease control (CK_1_, 2,099 OTUs), and the healthy control (CK_2_, 1,316 OTUs). In our previous report, 7,028 known OTUs were detected in citrus leaf midribs of the HLB-affected citrus treated by antibiotic combinations (PS and KO) in the field using the same PhyloChip™ G3 array. These OTUs were from 58 phyla, of which five contained 100 or more OTUs, *Proteobacteria* (44.1%), *Firmicutes* (23.5%), *Actinobacteria* (12.4%), *Bacteroidetes* (6.6%) and *Cyanobacteria* (3.2%). In the antibiotic treated samples, the number of OTUs decreased to a total of 5,599 [14]. A metagenomic analysis of citrus phloem alone showed that only the Las bacterium was associated with HLB [17]. Thus, these other families are most likely present in tissues other than the phloem and may relate to secondary proliferation in declining leaves rather than relating to initial HLB development.

Comparative analysis of microbial community provides an approach to understanding community structure and function. Some microorganisms isolated from plant tissues exhibit potential as biocontrol agents against phytopathogens [32], promote plant growth, and hasten plant development [33]. However, there are no reports of synergistic interactions between endophytic microorganisms and phytopathogens that result in a plant disease. In a pairwise comparison (Fig. 3, Fig. S1-B, Fig. S3-F, Fig. S5-J and Table S1), 23 OTUs from the family *Alcaligenaceae* were abundant only in the healthy control (CK_2_), including most OTUs of *Achromobacter xylosoxidans* (14378, 14510, 14570, 14691,14717, 14737, 14789, 15105, 15502, 15845 and 15854) and other *Achromobacter* spp. *A. xylosoxidans* has been reported to inhibit the growth of plant pathogens by the production of chitinase, or other inhibitory substance [32], [34], or through iron competition [35]. In a previous report, an increased abundance of *Alcaligenaceae* was reported in the asymptomatic samples when compared to the symptomatic samples of Las-infected citrus [15]. Due to the limited amount of soluble iron in the rhizosphere, microbes and plants scavenge for iron using highly sophisticated iron binding and uptake mechanisms [36]. The acquisition of iron is recognized as one of the key steps in the survival of any pathogen in its host [37]. Our results indicated Las-infected plants were deficient in zinc, iron, nitrogen, and phosphorus, and they produced more potassium and boron than uninfected plants (unpublished data). These findings may warrant further investigation on whether *A. xylosoxidans* plays a significant role in suppressing HLB disease symptoms.

The results (Fig. 3, Fig. S1-A, Fig. S2-D, Fig. S4-H and Table S1) presented here also indicated that 12 OTUs (59185, 59212, 59404, 59410, 59417, 59549, 59601, 59718, 59757, 59917, 59976 and 60144) from the genus *Methylobacterium* in the family of *Methylobacteriaceae* were more abundant in the disease control (CK_1_) when compared to the other treatments. *Methylobacterium* was also detected in the root samples from HLB-affected citrus plants [16]. The genus *Methylobacterium* resided in the xylem vessels of citrus plants, and abundant *Methylobacterium* spp. in citrus triggers CVC disease by a synergistic interaction with *X. fastidiosa* [31]. Therefore, the abundance of the endophytic *Methylobacterium* may be associated with HLB progression.

The antibacterial activity of an antibiotic is influenced by the state of bacterial responsiveness, the physicochemical environment at the infection site, and the degree of cooperation with the host defenses [38], [39]. The results presented here indicate that Amp but not Gm was effective in eliminating the Las bacterium. The grafted lemon scions had much more severe HLB symptoms with higher Las titers following Gm treatment (Table 1 and Fig. 2). Understanding the structure and species composition of bacterial communities is necessary for evaluating the influence of the applied antibiotics.

From a pharmacodynamic point, the intracellular concentration of the antibiotic is critical for Gm and the time of exposure is important for Amp. It is reported that Gm kills bacterial cells by inhibiting 30S ribosomal protein synthesis and disrupting lipopolysaccharides in the outer membrane [39]. Amp belongs to the penicillin group of betalactam antibiotics and acts as a competitive inhibitor of the transpeptidase to prevent bacterial cell wall synthesis in binary fission, which ultimately leads to bacterial cell lysis [40]. Ten abundant OTUs (16112, 16171, 16258, 16452, 16529, 16992, 17063, 17215, 17247, and 17254) from *Stenotrophomonas* spp. in the family of *Xanthomonadaceae* were detected only in the Amp-treatment, but not in the controls (Fig. 3A, Fig. S2-C, Fig. S3-D and Table S1). *Xanthomonadaceae* is a wide-spread family of bacteria belonging to the gamma subdivision of the Gram-negative proteobacteria, which includes two plant-pathogenic genera, *Xanthomonas* and *Xylella*, and the related genus *Stenotrophomonas*. *Stenotrophomonas* was abundant only in the Amp-treatment, and *Xylella* was not detected in any sample. It has been reported that *Stenotrophomonas* spp. produce antifungal antibiotics and have growth promoting activities on plants [41].

It is intriguing that the number and abundance of OTUs in the Gm-treatment were much more than those in the Amp-treatment and the controls. Over 85% of the total detected OTUs were found in the Gm-treatment. All the families with over 1% of the total OTUs in the Gm-treatment were abundant except *Lachnospiraceae* and *Ruminococcaceae* (Fig. 3B, Fig. S4-G and Fig. S5-I). However, Gm-treatment had lower percentage of *Actinobacteria* and *Cyanobacteria*. *Cyanobacteria* was reported to produce antimicrobial compounds against several Gram-positive bacteria, such as *Bacillus subtilis*, *Bacillus pumulis*, *Enterococcus faecalis*, *Staphylococcus aureus* and *Staphylococcus epidermidis*, and Gram-negative bacteria, such as *Escherichia coli*, *Pseudomonas aeruginosa* and *Klebsiella pneumoniae* [42]. We hypothesize that Gm-treatment might break the existing balance of bacterial communities in the citrus, and result in more OTUs detectible by killing or suppressing some critical OTUs in the balance. Further verification of this hypothesis is necessary to address this finding. Although an antibiotic treatment may be effective to a given number of bacterial diseases, it is critical to measure its ecological effects in addition to the effects on pathogens. In this research, we revealed the bacterial communities of citrus, with and without HLB infection, along with different antibiotic treatments, which has provided new insights into HLB progression, and the bases for the development of more effective and eco-friendly HLB control strategy.

### Availability of supporting data

The data sets supporting the results of this article are available in the Geo repository, GSE46728 http://www.ncbi.nlm.nih.gov/geo/query/acc.cgi?acc=GSE46728.

## Supporting Information

Figure S1 **Comparative trees of CK_1_ versus CK_2_.** Phylogenetic trees of families with over 1% of the total detected Operational Taxonomic Units (OTUs) from the bacterial community of leaf midribs of scions from grapefruit graft-inoculated with HLB-affected lemon scions (disease control, CK_1_) and with Las-free scions as the healthy controls (CK_2_). The half-circle **A**) OTUs present in CK_1_ and absent in CK_2_; **B**) OTUs present in CK_2_ and absent in CK_1_.
(DOCX)

Figure S2 **Comparative trees of Amp versus CK_1_.** Phylogenetic trees of families with over 1% of the total detected Operational Taxonomic Units (OTUs) from the bacterial community of leaf midribs from grapefruit graft-inoculated with HLB-affected lemon scions treated with ampicillin (Amp) and water (disease control, CK_1_). The half-circle **C**) OTUs present in Amp and absent in CK_1_; **D**) OTUs present in CK_1_ and absent in Amp.
(DOCX)

Figure S3 **Comparative trees of Amp versus CK_2_.** Phylogenetic trees of families with over 1% of the total detected Operational Taxonomic Units (OTUs) from the bacterial community of leaf midribs from grapefruit graft-inoculated with HLB-affected lemon scions treated with ampicillin (Amp) and with Las-free scions were the healthy controls (CK_2_). The half-circle **E**) OTUs present in CK_2_ and absent in mp; **F**) OTUs present in Amp and absent in CK_2_.
(DOCX)

Figure S4 **Comparative trees of Gm versus CK_1_.** Phylogenetic trees of families with over 1% of the total detected Operational Taxonomic Units (OTUs) from the bacterial community of leaf midribs from grapefruit graft-inoculated with HLB-affected lemon scions treated with gentamicin (Gm) and water (disease control, CK_1_). The half-circle **G**) OTUs present in Gm and absent in CK_1_; **H**) OTUs present in CK_1_ and absent in Gm.
(DOCX)

Figure S5 **Comparative trees of Gm versus CK_2_.** Phylogenetic trees of families with over 1% of the total detected Operational Taxonomic Units (OTUs) from the bacterial community of leaf midribs from grapefruit graft-inoculated with HLB-affected lemon scions treated with gentamicin (Gm) and with Las-free scions were the healthy controls (CK_2_). The half-circle **I**) OTUs present in CK_2_ and absent in Gm; **J**) OTUs present in Gm and absent in CK_2_.
(DOCX)

Table S1 Total number of Operational Taxonomic Units (OTUs) detected by PhyloChip™ G3 hybridization in leaf midribs from grapefruit graft-inoculated with *Ca.* L. asiaticus (Las)-free lemon scions (healthy control, CK_2_), and Las-infected lemon scions treated with ampicillin (Amp), gentamicin (Gm) and water (disease control CK_1_).
(DOCX)

## References

[1] Bové JM (2006) Huanglongbing: A destructive, newly-emerging, century-old disease of citrus. J Plant Pathol 88: 7–37.

[2] Donnua S, Paradornuwat A, Chowpongpang S, Thaveechai N (2012) Comparison between single and duplex conventional PCR for detection of ‘*Candidatus* Liberibacter asiaticus’, the causal agent of citrus Huanglongbing disease in Thailand. Crop Protection 41: 128–133.

[3] Meyer JM, Hoy MA, Boucias DG (2007) Morphological and molecular characterization of a *Hirsutella* species infecting the Asian citrus psyllid, *Diaphorina citri* Kuwayama (Hemiptera: Psyllidae), in Florida. J of Invert Pathol 95: 101–109. 10.1016/j.jip.2007.01.005 17382959

[4] Bové JM, Ayres AJ (2007) Etiology of three recent diseases ofcitrus in São Paulo State: Sudden death, variegated chlorosis and huanglongbing. IUBMB Life 59: 346–354. 17505974 10.1080/15216540701299326

[5] Gottwald TR (2010) Current epidemiological understanding of citrus huanglongbing. Ann Rev Phytopathol 48: 119–139. 20415578 10.1146/annurev-phyto-073009-114418

[6] Jagoueix S, Bové JM, Garnier M (1994) The phloem-limited bacterium of greening disease of citrus is a member of alpha subdivision of the Proteobacteria. Intl J Sys Bacteriol 44: 379–386. 10.1099/00207713-44-3-379 7520729

[7] Teixeira DC, Saillard C, Eveillard S, Danet JL, da Costa, et al (2005) ‘*Candidatus* Liberibacter americanus’, associated with citrus huanglongbing (greening disease) in São Paulo State, Brazil. Intl J Syst Evol Microbiol 55: 1857–1862. 10.1099/ijs.0.63677-0 16166678

[8] Hodges AW, Spreen TH (2012) Economic impacts of citrus greening (HLB) in Florida, 2006/7–2010/11. Electronic data information source (EDIS) update FE903 2012, University of Florida Department of Food and Resource Economics, University of Florida, Gainesville, FL. Available: http://news.ufl.edu/2012/01/24/greening-cost/.

[9] Aubert B (1990) Integrated activities for the control of huanglungbing-greening and its vector *Diaphorina* citri Kuwayama in Asia, In: Aubert B, TontyapornS, Buangsuwon D, editors. Rehabilitation of citrus industry in the Asia Pacific region, Proc. Asia Pacific Intern. Conf. on Citri Culture, Chiang Mai, Thailand, 4–10 Feb. 1990. pp. 133–44.

[10] Su HJ, Chang SC (1974) Electron microscopical study on the heat and tetracycline response, and ultra-structure of the pathogen complex causing citrus likubin disease. In: Proc. 8th Int. Congr. Electron Microscopy, Vol. 2., Canberra, Australia. pp. 628–629.

[11] Nariani TK (1981) Integrated approach to control citrus greening disease in India. Intl Soc Citriculture 1: 471–472.

[12] Zhang MQ, Powell CA, Guo Y, Doud MS, Duan YP (2012) A graft-based chemotherapy method for screening effective molecules and rescuing huanglongbing-affected citrus plants. Phytopathology 102: 567–574. 22568814 10.1094/PHYTO-09-11-0265

[13] Zhang MQ, Powell CA, Zhou LJ, He ZL, Stover E, et al (2011) Chemical compounds effective against the citrus Huanglongbing bacterium ‘*Candidatus* Liberibacter asiaticus’ in planta. Phytopathology 101: 1097–1103. 21834727 10.1094/PHYTO-09-10-0262

[14] Zhang MQ, Powell CA, Guo Y, Benyon L, Duan YP (2013) Characterization of the microbial community structure in ‘*Candidatus* Liberibacter asiaticus’-infected citrus plants treated with antibiotics in the field. BMC Microbiology 13: 112. 23701743 10.1186/1471-2180-13-112 PMC3672075

[15] Sagaram US, DeAngelis KM, Trivedi P, Andersen GL, Lu SE, et al (2009) Bacterial diversity analysis of Huanglongbing pathogen-infected citrus, using PhyloChip arrays and 16S rRNA gene clone library sequencing. Appl Environ Microbiol 75: 1566–1574. 19151177 10.1128/AEM.02404-08 PMC2655442

[16] Trivedi P, Spann T, Wang N (2011) Isolation and characterization of beneficial bacteria associated with citrus roots in Florida. Microb Ecol 62: 324–336. 21360139 10.1007/s00248-011-9822-y

[17] Tyler HL, Roesch LF, Gowda S, Dawson WO, Triplett EW (2009) Confirmation of the sequence of ‘*Candidatus* Liberibacter asiaticus’ and assessment of microbial diversity in Huanglongbing-infected citrus phloem using a metagenomic approach. Mol Plant Microbe Interact 22: 1624–1634. 19888827 10.1094/MPMI-22-12-1624

[18] Hazen TC, Dubinsky EA, DeSantis TZ, Andersen GL, Piceno YM, et al (2010) Deep-sea oil plume enriches indigenous oil degrading bacteria. Science 330: 204–208. 20736401 10.1126/science.1195979

[19] Li WB, Hartung JS, Levy L (2008) Optimized quantification of unculturable ‘*Candidatus* Liberibacter spp.’ in host plants using real-time PCR. Plant Dis 92: 854–861. 10.1094/PDIS-92-6-0854 30769724

[20] Gascuel O (1997) BIONJ: an improved version of the NJ algorithm based on a simple model of sequence data. Mol Biol Evol 14: 685–695. 9254330 10.1093/oxfordjournals.molbev.a025808

[21] Saitou N, Nei M (1987) The neighbor-joining method: a new method for reconstructing phylogenetic trees. Mol Biol Evol 4: 406–425. 3447015 10.1093/oxfordjournals.molbev.a040454

[22] Felsenstein J (1981) Evolutionary trees from DNA sequences: a maximum likelihood approach. J Mol Evol 17: 368–376. 7288891 10.1007/BF01734359

[23] Guindon S, Gascuel O (2003) A simple, fast, and accurate algorithm to estimate large phylogenies by maximum likelihood. Syst Biol 52: 696–704. 14530136 10.1080/10635150390235520

[24] Letunic I, Bork P (2006) Interactive Tree Of Life (iTOL): an online tool for phylogenetic tree display and annotation. Bioinformatics 23: 127–128. 17050570 10.1093/bioinformatics/btl529

[25] Letunic I, Bork P (2011) Interactive Tree Of Life v2: online annotation and display of phylogenetic trees made easy. Nucl Acids Res 39: W475–W478. 21470960 10.1093/nar/gkr201 PMC3125724

[26] Costa LEO, Queiroz MV, Borges AC, Moraes CA, Araújo EF (2012) Isolation and characterization of endophytic bacteria isolated from the leaves of the common bean (*P. vulharis*). Br J Microbiol 43: 1562–1575. 10.1590/S1517-838220120004000041 PMC3769033 24031988

[27] Amann R, Ludwig W, Schleifer K (1995) Phylogenetic identification and in situ detection of individual microbial cells without cultivation. Microbiol Rev59: 143–169. 10.1128/mr.59.1.143-169.1995 PMC239358 7535888

[28] Kell DB, Kaprelyants AS, Weichart DH, Harwood CR, Barer MR (1998) Viability and activity in readily culturable bacteria: a review and discussion of the practical issues. Antonie Van Leeuwenhoek 73: 169–187. 9717575 10.1023/a:1000664013047

[29] Probst A, Vaishampayan P, Osman S, Moissl-Eichinger C, Andersen GL, et al (2010) Microbial diversity of anaerobes from spacecraft assembly clean rooms. Appl Environ Microbiol 76: 2837–2845. 20228115 10.1128/AEM.02167-09 PMC2863428

[30] Kaprelyants AS, Gottschal JC, Kell DB (1993) Dormancy in non-sporulating bacteria. FEMS Microbiol Rev 104: 271–286. 10.1111/j.1574-6968.1993.tb05871.x 8318260

[31] Araújo W, Marcon J, Maccheroni W Jr, Elsas JD, Vuurde JWL, et al (2002) Diversity of endophytic bacterial populations and their interaction with *Xylella fastidiosa* in citrus plants. Appl Environ Microbiol 68: 4906– 4914. 12324338 10.1128/AEM.68.10.4906-4914.2002 PMC126398

[32] Vaidya RJ, Shah IM, Vyas PR, Chhatpar HS (2001) Production of chitinase and its optimization from a novel isolate *Alcaligenes xylosoxydans*: potential in antifungal biocontrol. World J Microbiol Biotechnol 17: 691–696.

[33] Lodewyckx C, Vangronsveld J, Porteus F, Moore ERB, Taghavi S, et al (2002) Endophytic bacteria and their potential applications. Crit Rev Plant Sci 21: 586–606.

[34] Yan PS, Song Y, Sakuno E, Nakajima H, Nakagawa H, et al (2004) Cyclo (L-Leucyl-L-Prolyl) produced by *Achromobacter xylosoxidans* inhibits aflatoxin production by *Aspergillus parasiticus* . Appl Environ Microbiol 70: 7466–7473. 15574949 10.1128/AEM.70.12.7466-7473.2004 PMC535151

[35] Yuen GY, Schroth MN (1986) Inhibition of *Fusarium oxysporum* f. sp. dianthi by iron competition with an *Alcaligenes* sp. Phytopathology 76: 171–176.

[36] Moretti M, Gilardi G, Gullino ML, Garibaldi A (2008) Biological control potential of *Achromobacter xylosoxydans* for suppressing *Fusarium* wilt of tomato. Intl J Bot 4: 369–375.

[37] Colin R, Lynn GD (2000) Iron metabolism in pathogenic bacteria. Ann Rev Microbiol 54: 881–941. 11018148 10.1146/annurev.micro.54.1.881

[38] Barcia-Macay M, Seral C, Mingeot-Leclercq MP, Tulkens PM, Van Bambeke F (2006) Pharmacodynamic evaluation of the intracellular activities of antibiotics against Staphylococcus aureus in a model of THP-1 macrophages. Antimicrob Agents Chemother 50: 841–851. 16495241 10.1128/AAC.50.3.841-851.2006 PMC1426441

[39] Van Bambeke F, Barcia-Macay M, Lemaire S, Tulkens PM (2006) Cellular pharmacodynamics and pharmacokinetics of antibiotics: current views and perspectives. Curr Opin Drug Discov Devel 9: 218–230. 16566292

[40] Blumberg PM, Stromniger JL (1974) Interaction of penicillin with the bacterial cell: penicillin-binding proteins and penicillin-sensitive enzymes. Bacteriol Rev 38: 291–335. 4608953 10.1128/br.38.3.291-335.1974 PMC413858

[41] Wolf A, Fritze A, Hagemann M, Berg G (2002) *Stenotrophomonas rhizophila* sp. nov., a novel plant-associated bacterium with antifungal properties. Intl J Syst Evol Microbiol 52: 1937–1944. 10.1099/00207713-52-6-1937 12508851

[42] Heidari F, Riahi H, Yousefzadi M, Asadi M (2012) Antimicrobial activity of *cyanobacteria* isolated from hot spring of geno. Middle-East J Sci Res 12: 336–339.

