# Supplementary material for: Deciphering the Bacterial Microbiome of Citrus Plants in Response to ‘Candidatus Liberibacter asiaticus’-Infection and Antibiotic Treatments
Source: PLoS One. 2013 Nov 8;8(11):e76331. doi: 10.1371/journal.pone.0076331 (PMC3826729; doi:10.1371/journal.pone.0076331)
Supplement: Figure S1 — Comparative trees of CK1 versus CK2. Phylogenetic trees of families with over 1% of the total detected Operational Taxonomic Units (OTUs) from the bacterial community of leaf midribs of scions from grapefruit graft-inoculated with HLB-affected lemon scions (disease control, CK1) and with Las-free scions as the healthy controls (CK2). The half-circle A) OTUs present in CK1 and absent in CK2; B) OTUs present in CK2 and absent in CK1. (DOCX) [file pone.0076331.s001.docx]

Fig. S1. Phylogenetic trees of families with over 1% of the total detected Operational Taxonomic Units (OTUs) from the bacterial community of leaf midribs from grapefruit graft-inoculated with HLB-affected lemon scions (disease control, CK_1_) and with Las-free scions as the healthy controls (CK_2_). The half-circles indicate: A, OTUs present in CK_1_ and absent in CK_2_; B, OTUs present in CK_2_ and absent in CK_1_.
