# Supplementary material for: Deciphering the Bacterial Microbiome of Citrus Plants in Response to ‘Candidatus Liberibacter asiaticus’-Infection and Antibiotic Treatments
Source: PLoS One. 2013 Nov 8;8(11):e76331. doi: 10.1371/journal.pone.0076331 (PMC3826729; doi:10.1371/journal.pone.0076331)
Supplement: Figure S2 — Comparative trees of Amp versus CK1. Phylogenetic trees of families with over 1% of the total detected Operational Taxonomic Units (OTUs) from the bacterial community of leaf midribs from grapefruit graft-inoculated with HLB-affected lemon scions treated with ampicillin (Amp) and water (disease control, CK1). The half-circle C) OTUs present in Amp and absent in CK1; D) OTUs present in CK1 and absent in Amp. (DOCX) [file pone.0076331.s002.docx]

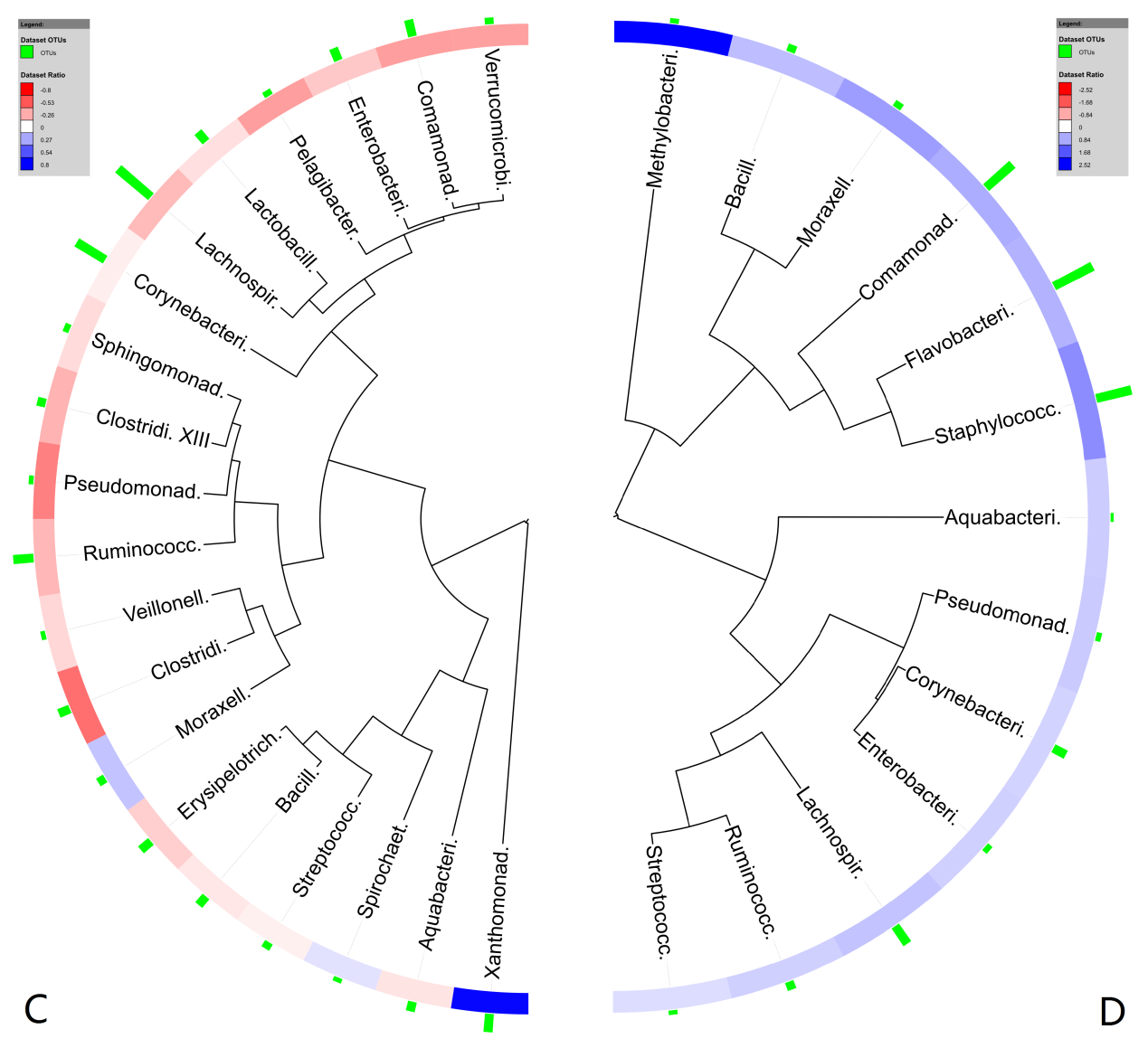


**Amp *vs* CK*_1_***

**Fig. S2.** Phylogenetic trees of families with over 1% of the total detected Operational Taxonomic Units (OTUs) from the bacterial community of leaf midribs from grapefruit graft-inoculated with HLB-affected lemon scions treated with ampicillin (Amp) and water (disease control, CK_1_). The half-circles indicate: **C**, OTUs present in Amp and absent in CK_1_; **D**, OTUs present in CK_1_ and absent in Amp.
