# Supplementary material for: Deciphering the Bacterial Microbiome of Citrus Plants in Response to ‘Candidatus Liberibacter asiaticus’-Infection and Antibiotic Treatments
Source: PLoS One. 2013 Nov 8;8(11):e76331. doi: 10.1371/journal.pone.0076331 (PMC3826729; doi:10.1371/journal.pone.0076331)
Supplement: Figure S3 — Comparative trees of Amp versus CK2. Phylogenetic trees of families with over 1% of the total detected Operational Taxonomic Units (OTUs) from the bacterial community of leaf midribs from grapefruit graft-inoculated with HLB-affected lemon scions treated with ampicillin (Amp) and with Las-free scions were the healthy controls (CK2). The half-circle E) OTUs present in CK2 and absent in mp; F) OTUs present in Amp and absent in CK2. (DOCX) [file pone.0076331.s003.docx]

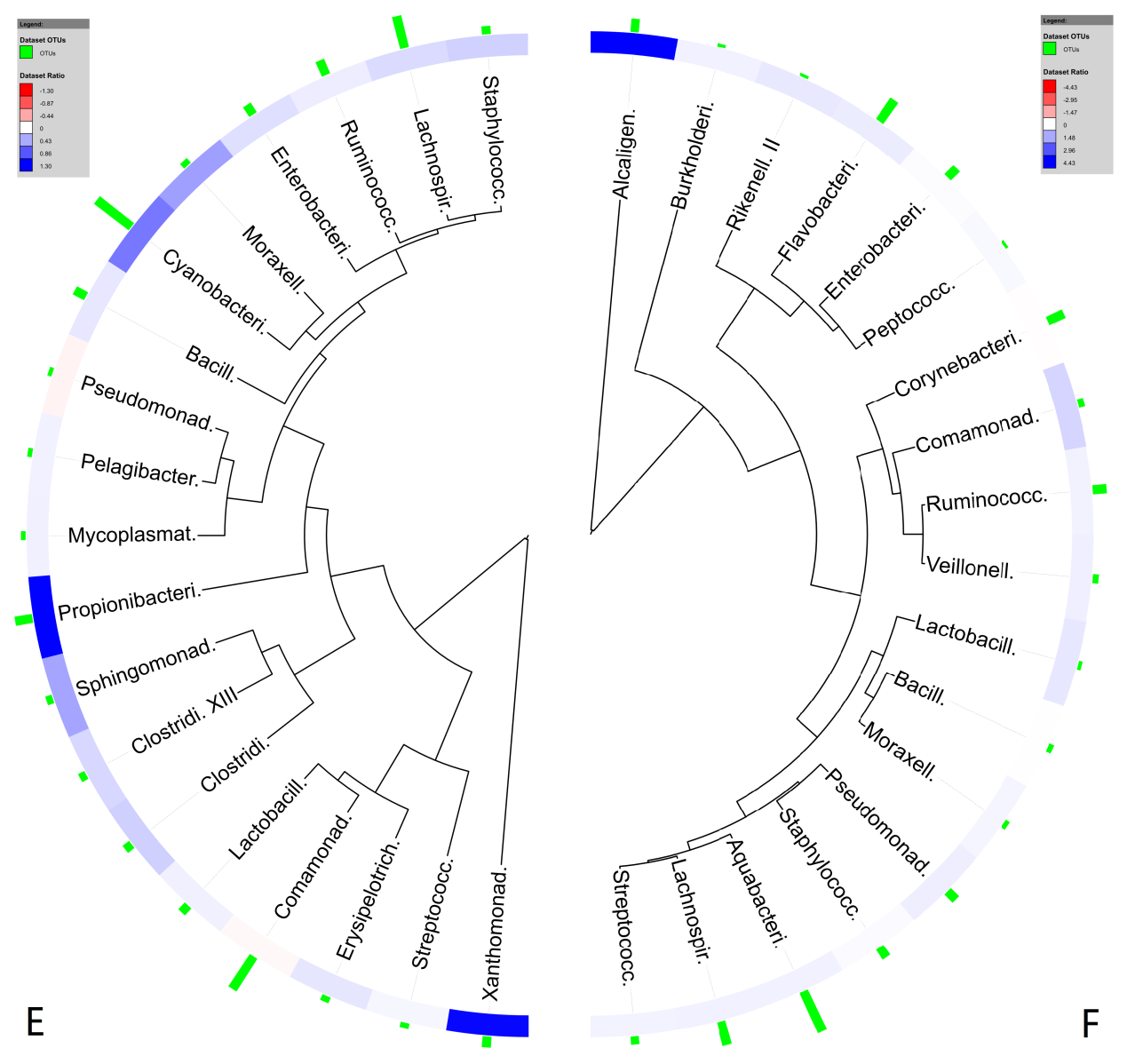


**Amp *vs* CK*_2_***

**Fig. S3.** Phylogenetic trees of families with over 1% of the total detected Operational Taxonomic Units (OTUs) from the bacterial community of leaf midribs from grapefruit graft-inoculated with HLB-affected lemon scions treated with ampicillin (Amp) and with Las-free scions were the healthy controls (CK_2_). The half-circles indicate: **E**, OTUs present in Amp and absent in CK_2_; **F**, OTUs present in CK_2_ and absent in Amp.
