# Supplementary material for: Deciphering the Bacterial Microbiome of Citrus Plants in Response to ‘Candidatus Liberibacter asiaticus’-Infection and Antibiotic Treatments
Source: PLoS One. 2013 Nov 8;8(11):e76331. doi: 10.1371/journal.pone.0076331 (PMC3826729; doi:10.1371/journal.pone.0076331)
Supplement: Figure S4 — Comparative trees of Gm versus CK1. Phylogenetic trees of families with over 1% of the total detected Operational Taxonomic Units (OTUs) from the bacterial community of leaf midribs from grapefruit graft-inoculated with HLB-affected lemon scions treated with gentamicin (Gm) and water (disease control, CK1). The half-circle G) OTUs present in Gm and absent in CK1; H) OTUs present in CK1 and absent in Gm. (DOCX) [file pone.0076331.s004.docx]

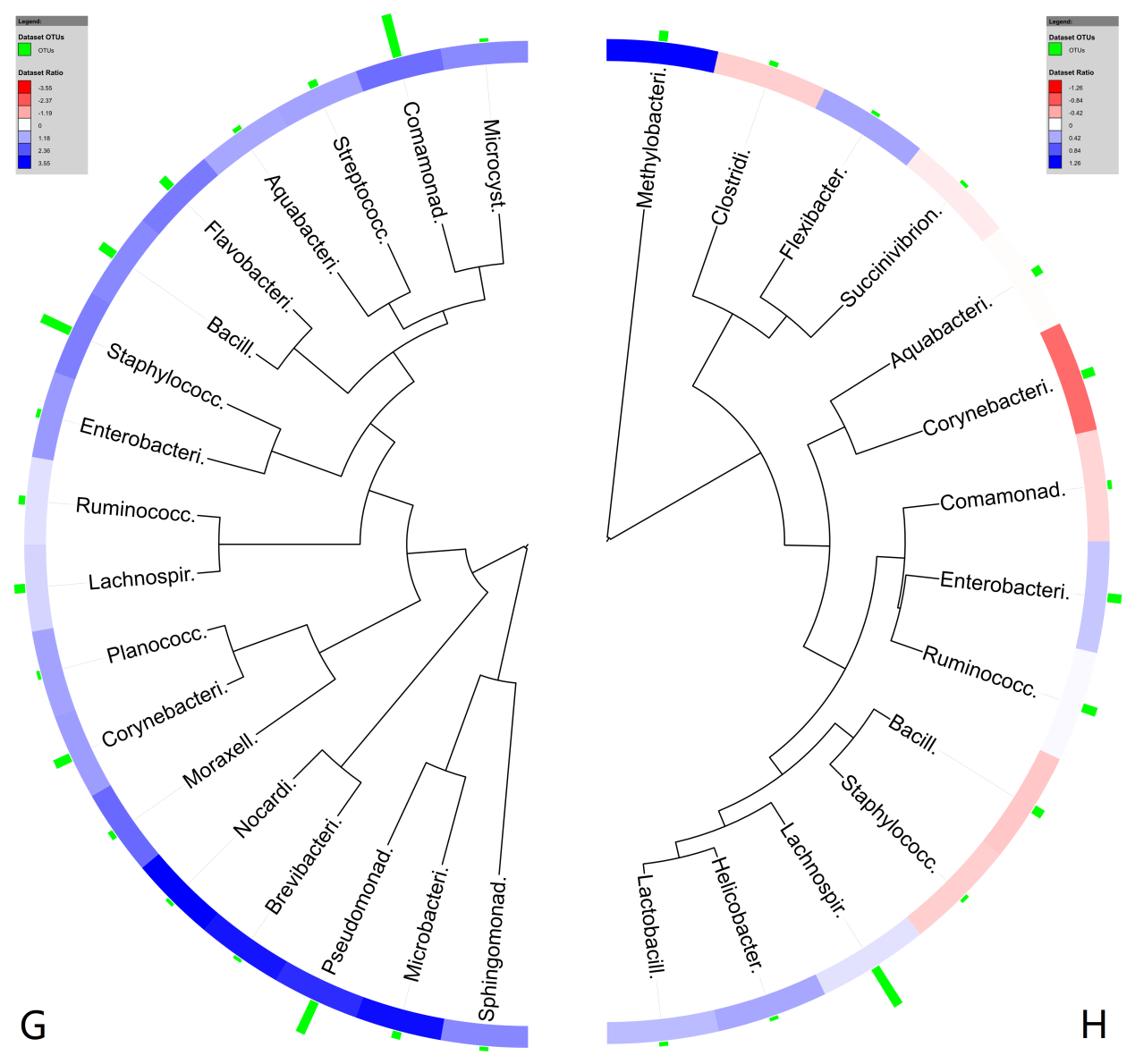


**Gm *vs* CK_1_**

**Fig. S4.** Phylogenetic trees of families with over 1% of the total detected Operational Taxonomic Units (OTUs) from the bacterial community of leaf midribs from grapefruit graft-inoculated with HLB-affected lemon scions treated with gentamicin (Gm) and water (disease control, CK_1_). The half-circles indicate: **G**, OTUs present in Gm and absent in CK_1_; **H**, OTUs present in CK_1_ and absent in Gm.
