# Supplementary material for: Deciphering the Bacterial Microbiome of Citrus Plants in Response to ‘Candidatus Liberibacter asiaticus’-Infection and Antibiotic Treatments
Source: PLoS One. 2013 Nov 8;8(11):e76331. doi: 10.1371/journal.pone.0076331 (PMC3826729; doi:10.1371/journal.pone.0076331)
Supplement: Figure S5 — Comparative trees of Gm versus CK2. Phylogenetic trees of families with over 1% of the total detected Operational Taxonomic Units (OTUs) from the bacterial community of leaf midribs from grapefruit graft-inoculated with HLB-affected lemon scions treated with gentamicin (Gm) and with Las-free scions were the healthy controls (CK2). The half-circle I) OTUs present in CK2 and absent in Gm; J) OTUs present in Gm and absent in CK2. (DOCX) [file pone.0076331.s005.docx]

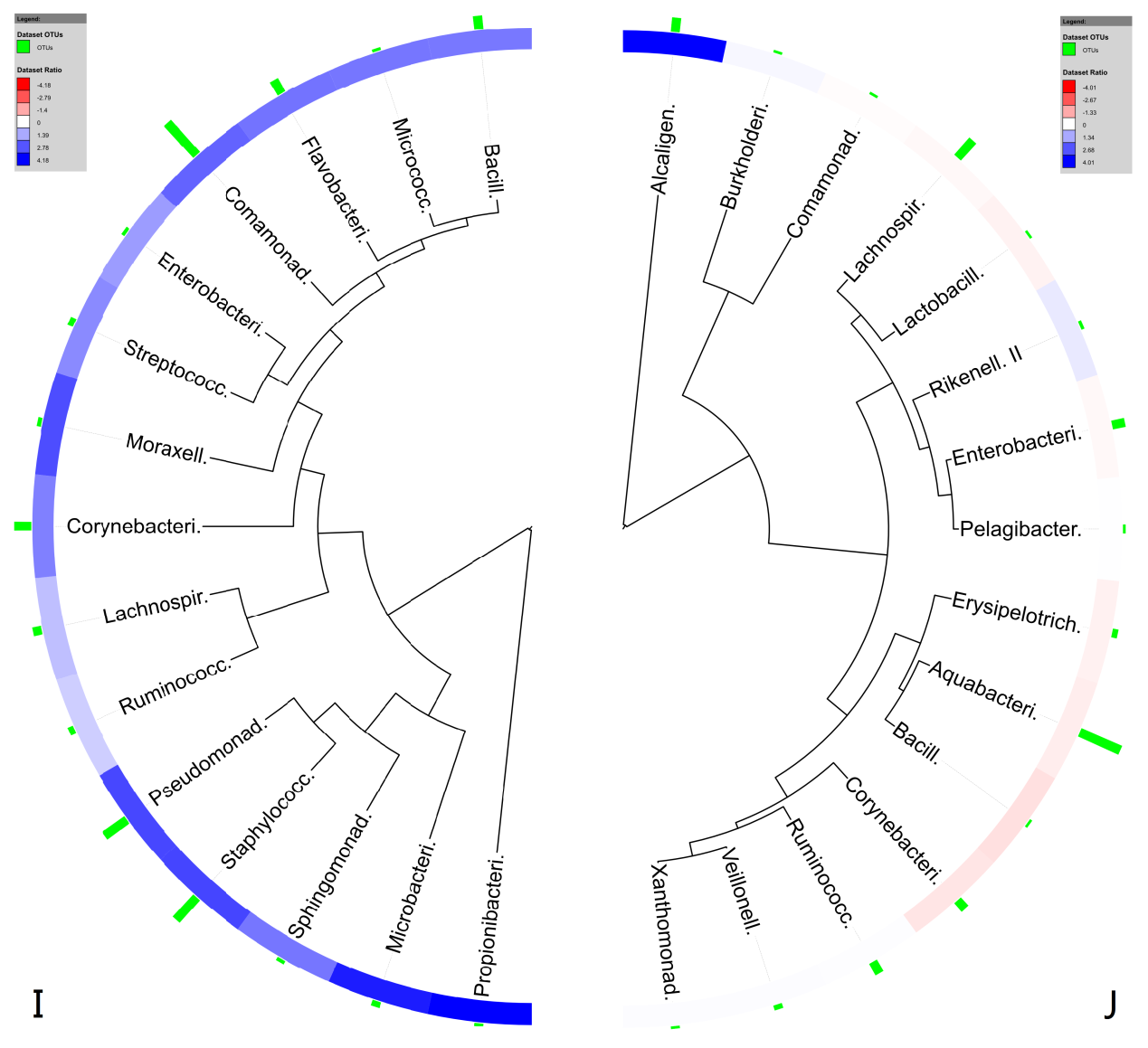


**Gm *vs* CK_2_**

**Fig. S5.** Phylogenetic trees of families with over 1% of the total detected Operational Taxonomic Units (OTUs) from the bacterial community of leaf midribs from grapefruit graft-inoculated with HLB-affected lemon scions treated with gentamicin (Gm) and with Las-free scions were the healthy controls (CK_2_). The half-circles indicate: **I**, OTUs present in Gm and absent in CK_2_; **J**, OTUs present in CK_2_ and absent in Gm.
