# Supplementary material for: Deciphering the Bacterial Microbiome of Citrus Plants in Response to ‘Candidatus Liberibacter asiaticus’-Infection and Antibiotic Treatments
Source: PLoS One. 2013 Nov 8;8(11):e76331. doi: 10.1371/journal.pone.0076331 (PMC3826729; doi:10.1371/journal.pone.0076331)
Supplement: Table S1 — Total number of Operational Taxonomic Units (OTUs) detected by PhyloChip™ G3 hybridization in leaf midribs from grapefruit graft-inoculated with Ca. L. asiaticus (Las)-free lemon scions (healthy control, CK2), and Las-infected lemon scions treated with ampicillin (Amp), gentamicin (Gm) and water (disease control CK1). (DOCX) [file pone.0076331.s006.docx]

Table S1. Total number of Operational Taxonomic Units (OTUs) detected by PhyloChip™ G3 hybridization in leaf midribs of scions from graft-inoculated with *Ca.* L. asiaticus (Las)-free lemon scions (healthy control, CK_2_), and Las-infected lemon scions treated with ampicillin (Amp), gentamicin (Gm) or water (disease control CK_1_).

| Phylum | Class | Order | Family | Amp | Gm | CK_2_ | CK_1_ | Total |
| --- | --- | --- | --- | --- | --- | --- | --- | --- |
| *Acidobacteria* | |  |  | 24 | 47 | 14 | 22 | 67 |
| *Actinobacteria* |  | |  | 416 | 1112 | 249 | 413 | 1189 |
|  |  |  | *Actinosynnemataceae* | 3 | 22 | 0 | 3 | 23 |
|  |  |  | *Coriobacteriaceae* | 9 | 17 | 7 | 11 | 21 |
|  |  |  | *Corynebacteriaceae* | 281 | 517 | 206 | 278 | 557 |
|  |  |  | *Microthrixaceae* | 20 | 192 | 7 | 19 | 201 |
|  |  |  | *Nocardiopsaceae* | 9 | 94 | 3 | 5 | 94 |
|  |  |  | *Propionibacteriaceae* | 46 | 61 | 3 | 47 | 62 |
|  |  |  | Other | 48 | 209 | 23 | 50 | 231 |
| *Bacteroidetes* | |  |  | 53 | 424 | 80 | 198 | 461 |
|  | *Bacteroidia* | |  | 18 | 38 | 18 | 18 | 54 |
|  | *Flavobacteria* | |  | 16 | 355 | 54 | 167 | 362 |
|  | *Sphingobacteria* | |  | 19 | 31 | 8 | 13 | 45 |
| *Chloroflexi* | |  |  | 24 | 33 | 18 | 21 | 49 |
| *Cyanobacteria* | |  |  | 107 | 125 | 94 | 107 | 169 |
| *Fibrobacteres* | |  |  | 3 | 9 | 5 | 3 | 9 |
| *Firmicutes* | |  |  | 443 | 1825 | 297 | 558 | 2147 |
|  | *Bacilli* |  |  | 175 | 1298 | 125 | 302 | 1373 |
|  | *Clostridia* | |  | 268 | 527 | 173 | 256 | 774 |
| *Proteobacteria* | |  |  | 501 | 2465 | 418 | 603 | 2870 |
|  | *Alphaproteobacteria* | | | 84 | 321 | 49 | 102 | 405 |
|  |  | *Caulobacterales* | | 2 | 26 | 2 | 5 | 29 |
|  |  | *Rhizobials* | | 27 | 114 | 19 | 48 | 154 |
|  |  | *Rhodobacterales* | | 6 | 21 | 3 | 4 | 28 |
|  |  | *Rhodospirillales* | | 15 | 52 | 12 | 19 | 57 |
|  |  | *Rickettsiales* | | 15 | 30 | 9 | 14 | 49 |
|  |  | *Sphingomonadale* | | 19 | 74 | 3 | 11 | 84 |
|  |  | Other |  | 0 | 4 | 1 | 1 | 4 |
|  | *Betaproteobacteria* | | | 195 | 1125 | 204 | 284 | 1256 |
|  |  |  | *Aquabacteriaceae* | 52 | 101 | 124 | 47 | 179 |
|  |  |  | *Comamonadaceae* | 114 | 869 | 29 | 210 | 880 |
|  |  |  | other | 29 | 155 | 51 | 27 | 197 |
|  | *Deltaproteobacteria* | | | 41 | 96 | 32 | 45 | 126 |
|  | *Epsilonproteobacteria* | | | 12 | 20 | 9 | 10 | 32 |
|  | *Gammaproteobacteria* | | | 169 | 902 | 124 | 162 | 1052 |
|  |  |  | *Enterobacteriaceae* | 35 | 73 | 29 | 35 | 121 |
|  |  |  | *Moraxellaceae* | 20 | 103 | 13 | 31 | 113 |
|  |  |  | *Pseudomonadaceae* | 19 | 552 | 29 | 29 | 557 |
|  |  |  | *Xanthomonadaceae* | 30 | 33 | 6 | 6 | 62 |
|  |  |  | other | 65 | 141 | 47 | 61 | 199 |
| *Spirochaetes* | |  |  | 17 | 36 | 7 | 10 | 45 |
| *Tenericutes* | |  |  | 55 | 68 | 37 | 44 | 97 |
| Other |  |  |  | 152 | 212 | 97 | 120 | 304 |
| **Total** |  |  |  | **1795** | **6356** | **1306** | **2099** | **7407** |
